# Penny Wisdom—Mistaken Philanthropy

**Published:** 1897-03

**Authors:** 


PENNY WISDOM: MISTAKEN PHILANTHROPY.—There is no estimating the cost to the State of taking care of its defectives of all classes. They are as tenderly cared for as if their lives were the most precious, and an almost pious regard is had for their posterity. We spend millions to save the lives of waifs, insane, deaf and dumb and blind, and the criminal classes, seemingly without a though that they are multiplying, and that the next generation, and the next, and the next for all time to come, will be taxed still further for the care and maintanance of the progeny of those now the object of our tender solicitude.

We see by the report of the Illinois Board of State Commis sioners of Public Charities, that for the last biennal term the State appropriated for these charities $2,962,550, and the report states: "This is a large amount, yet it is only one factor in the incalculable sum which the State, by private and public contribution devotes to the care of the dependent classes."

And so with Texas and other States. Why is not some step taken to diminish the supply, to cut off the stream of defectives that pours in on taxpayers in an ever widening volume? As Dr. Daniel said in a paper on this subject: "Sisyphus condemned to eternally roll the stone, had no more hopeless, endless task than we are now engaged in: nor the Danaides one more impossible of accomplishment; it is as irrational as the attempt to purify a sewer by throwing disinfectants into the outlet. The helpless, worthless, vicious and dangerous come faster than love, philanthropy, religion, science and law can cure, care for, reform or dispose of them." In the name of humanity the multiplication of the unfit should be prevented.

